# Body-Surface-Area Related Renal Volume: A Common Normal Range from Birth to Adulthood

**DOI:** 10.6064/2012/949164

**Published:** 2012-06-17

**Authors:** Th. Scholbach, D. Weitzel

**Affiliations:** ^1^Klinik für Kinder- und Jugendmedizin, Städtischen Klinikum, Chemnitz gGmbH, Flemmingstraße 4, 09116 Chemnitz, Germany; ^2^Fachbereich Kinderheilkunde und Jugendmedizin, Deutsche Klinik für Diagnostik, Aukamm Allee 33, 65191 Wiesbaden, Germany

## Abstract

Renal volume is an important parameter of renal development. Deviations from normal volume may indicate pathologic conditions. Thus, during childhood, the ever changing renal volumes require the continuous referral to normal volume charts in order to classify actual volumes, which is rather inconvenient. In daily practice this is frequently disregarded and kidneys are evaluated by their appearance only. Therefore, we tested the hypothesis that body surface area (BSA) and renal volume grow proportionally from birth to adulthood. We divided the renal volume of a child by its BSA to get the BSA-related renal volume (BSARV) and found no differences between left and right kidneys and a normal distribution for all kidneys regardless of the patient's age. BSARV has a common normal range for all age groups with the 10th percentile of 45 and the 90th percentile of 85 mL/m^2^. 80% of all kidneys do not exceed the volume of their counterparts by more than 20%. BSARV alleviates the correct evaluation of a child's renal volume regardless of age and reveals pathological influences by the simple observation that a kidney deviates from a former percentile or *z*-value. This is especially valuable in the followup of kidneys with chronic diseases.

## 1. Introduction

Renal volume (RV) grows throughout fetal development [1–3] and childhood [4–8]. Therefore, the evaluation of the renal volume is crucial to assess a normal renal development as well as to detect renal diseases. To evaluate an individual's renal size, it is therefore necessary to compare it with age-related normal value charts [4], percentiles [9], or nomograms [10]. On the one hand, this is cumbersome and therefore often disregarded. On the other hand, the referral to age groups is unphysiological, because it is not logical to assume, that normal ranges do jump if a certain child crosses the limits of its age group. At present, the higher age group requires then the reevaluation of the actual renal volume with the subsequent normal value chart for this age group.

We hypothesize that the function of the kidneys meets the metabolic requirements of the whole organism. These metabolic requirements are correlated with the body surface area (BSA) [11, 12].

## 2. Aim

The aim of the present study was therefore to investigate if a correlation of BSA and renal volumes exists irrespective to patients' age. By dividing renal volume by BSA we aimed to eliminate the tendency of both parameters to change with preadult growth.

## 3. Material and Methods

### 3.1. Patients

Our patients stemmed from a population of newborn and babies who were recruited for a renal ultrasound screening program in Hesse and from children from 1 month to 18 years of age from a tertiary ultrasound outpatient department in Saxony. From these children, we selected randomly 624 children, equally distributed to 5 age groups, with normal renal morphology, empty renal pelvis, undilated ureters and without anamnestical hints to renal disease (<3 m; <3 y; <6 y; <12 y; <18 y with *N* = 123; 109; 138; 127; 127, resp.). 

### 3.2. BSA Calculation

BSA was calculated according to D. Du Bois and E. F. Du Bois [13] as

(1) BSA[m2]  =body  weight  [kg]0.425∗body  height  [cm]0.725∗0.007184.

#### 3.2.1. Calculation of BSA-Related Renal Volume (BSARV)

Renal volume (RV) was calculated according to the volume formula of a rotational ellipsoid [4, 14, 15]:

(2) RV[mL]=L∗W∗D∗π6,

where *L*—length, *W*—width and *D*—depth.

 All kidney dimensions are maximum values of strictly longitudinal and transverse sections through the centre of the kidney and were recorded from a dorsal approach with the patient lying in a prone position.

BSARV was then calculated as

(3) BSARV=RV(mL)BSA(m2).

The relative renal volume was defined as the ratio of left or right renal volume divided by the total renal volume.

### 3.3. Statistics

The renal volume data were evaluated with respect to a normal distribution with the Kolmogorov-Smirnov test (*K*-*S* test).

Differences between groups were examined with the Mann-Whitney-*U*-test with a significance level of *P* < 0.05. 

## 4. Results

No significant differences were found between left and right renal volumes (*P* = 0.509).

**Figure 1:**
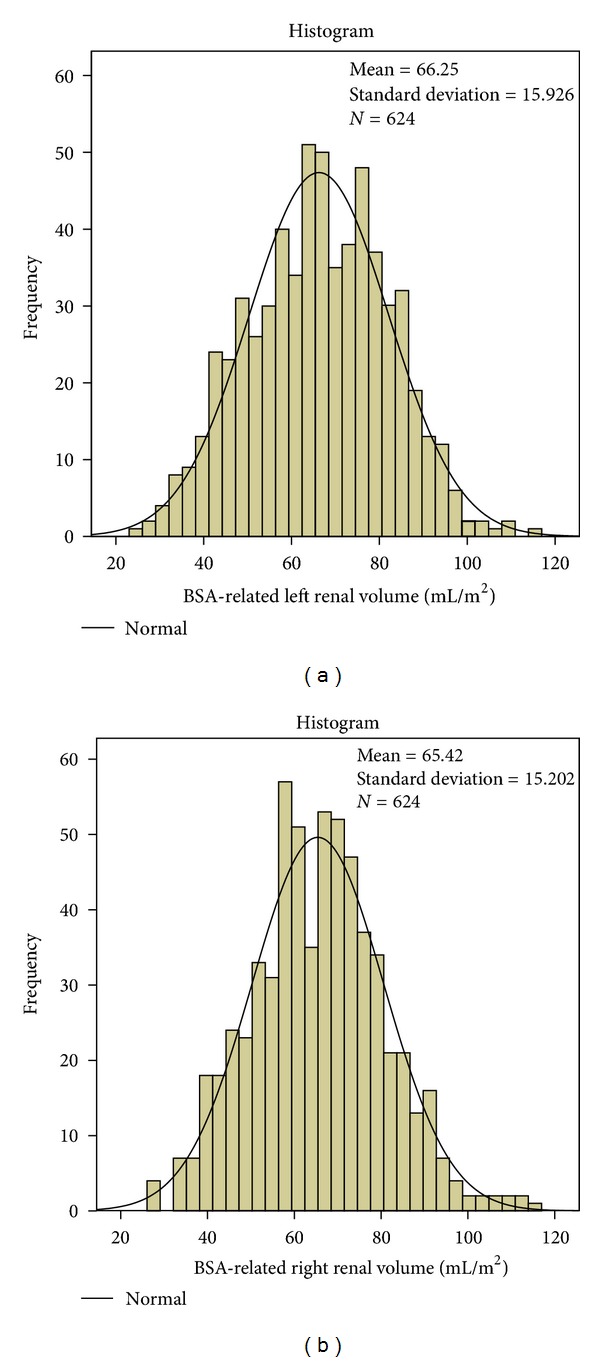
(a) Normal distribution of BSARV of the left kidney: mean = 66.25 mL/m², *N* = 624, SD = 15.93. (b) Normal distribution of BSARV of the right kidney: mean = 65.55 mL/m², *N* = 624, SD = 15.20.

**Figure 2:**
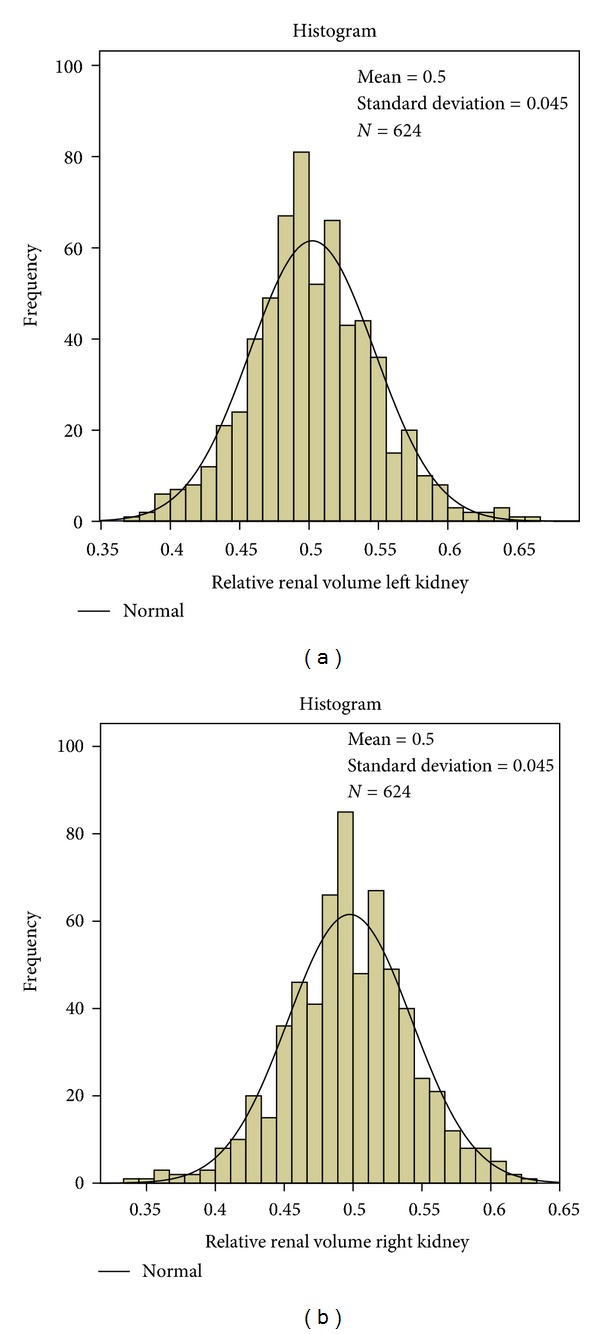
(a) Normal distribution of relative renal volume of the left kidney: mean = 0.50, *N* = 624, SD = 0.045. (b) Normal distribution of relative renal volume of the right kidney: mean = 0.50, *N* = 624. SD = 0.045.

BSARV is normally distributed irrespective of body side and body size from birth to 18 years (*P* = 0.822 and 0.727 for left and right BSARV, resp., tested with the *K*-*S* test) (Figures 1(a) and 1(b)). The common normal range for both kidneys ranges from 45 mL/m² (10th percentile) to 85 mL/m² (90th percentile) with a mean value of 66 mL/m² (Table 1). A strong symmetry is found between left and right kidneys with the expected normal distribution of the relative renal volumes of both sides and nearly equal percentiles (Table 1; lower rows). The normal range of the relative renal volume lies between the 10th percentile of 0.45 and the 90th percentile of 0.55 (Figures 2(a) and 2(b)) with a mean of 0.50 and a standard deviation of 0.045.

No significant differences were found between BSA-related left and right renal volumes of girls and boys in the entire population as well as in the five age groups (Table 2).

## 5. Discussion

The examination of a kidney's size may be a clue to renal function [16]. Volume measurements may predict single renal glomerular filtration rate better than renal length measurements [17]. Fetal renal growth was found predictive for intrauterine growth retardation and the 10th percentile of renal volume was found to be the best cutoff value [18]. For practical reasons, with respect to the inherent difficulties to investigate young children, we recommend to regard a range from 10 to 90% of the normally distributed BSARV as a normal range for all children and adolescents. As early as two decades ago, attempts were made to correlate renal function to parenchymal volume in urograms [19]. 

In renal sonograms, renal volume correlated better than volumes of the central echogenic area and the renal parenchyma with height, weight, and total body area in adults [20]. Nevertheless, the differentiation of total renal volumes and renal pelvis volumes might help to unveil very early damaging influences of the renal development as demonstrated in an MRI study [21]. Three-dimensional ultrasound, however, does not yet meet the needs of daily practice in readily differentiating parenchyma from renal pelvis [22]. Future developments might ease automated 3D rendering of renal parenchyma and its differentiation from surrounding fatty and connective tissue, which both exhibit an echogenicity that is similar to the young child's renal parenchyma.

The new parameter of BSARV has some major advantages over existing parameters to evaluate renal volume.

Firstly, it makes the many separate normal charts unnecessary and combines them to an easy to remember two number range: 45–85 (mL/m²)-irrespective of age, sex, and body size.

Secondly, a relative renal volume greater than 55% of the total renal volume or less than 45%, or roughly a difference of both renal volume greater than 20%, should prompt the suspicion of a one-sided affection.

Thirdly, a BSA-related renal volume overwhelms the artificial virtual breaks in the followup of an individual kidney development, which emanate from changing from one to the next normal value chart, when the child grows up. 

Fourthly, each child can be followed up smoothly according to his individual percentile corridor, which, as with body length and weight, describes the individual prognosis of the kidney volume development. Derangements as swelling or growth delay, which are often encountered early in chronic renal disease, can be unmasked easily. 

All this promises a true advantage for children with renal disease by precocious detection of creeping renal volume changes. Therefore we recommend BSARV calculations and the observation of the relative renal volumes for all renal sonographic examinations in children and adolescents. 

## Figures and Tables

**Table 1:** Percentiles of BSARV and relative renal volumes of both kidneys.

	BSARV (mL/m² BSA)								
Percentiles	3%	5%	10%	25%	50%	75%	90%	95%	97%
Left kidney	36	40	45	55	66	78	86	92	94
Right kidney	38	40	45	55	66	75	85	90	94
Relative renal volume left kidney	0.42	0.43	0.45	0.47	0.50	0.53	0.56	0.58	0.59
Relative renal volume right kidney	0.41	0.42	0.44	0.47	0.50	0.53	0.55	0.57	0.58

**Table 2:** No significant differences of BSA-related renal volumes between both sexes (Mann-Whitney-*U*-test).

*P* values (Mann-Whitney *U* test) for differences of BSA-related renal volumes between both sexes		
	BSA-related left renal volume (mL/m²)	BSA-related right renal volume (mL/m²)
All age groups	0.712	0.973
Age < 3 m	0.480	0.152
Age > 3 m and ≤3 years	0.232	0.469
Age > 3 years and ≤6 years	0.074	0.327
Age > 6 years and ≤12 years	0.292	0.654
Age > 12 years and ≤18 years	0.589	0.285
